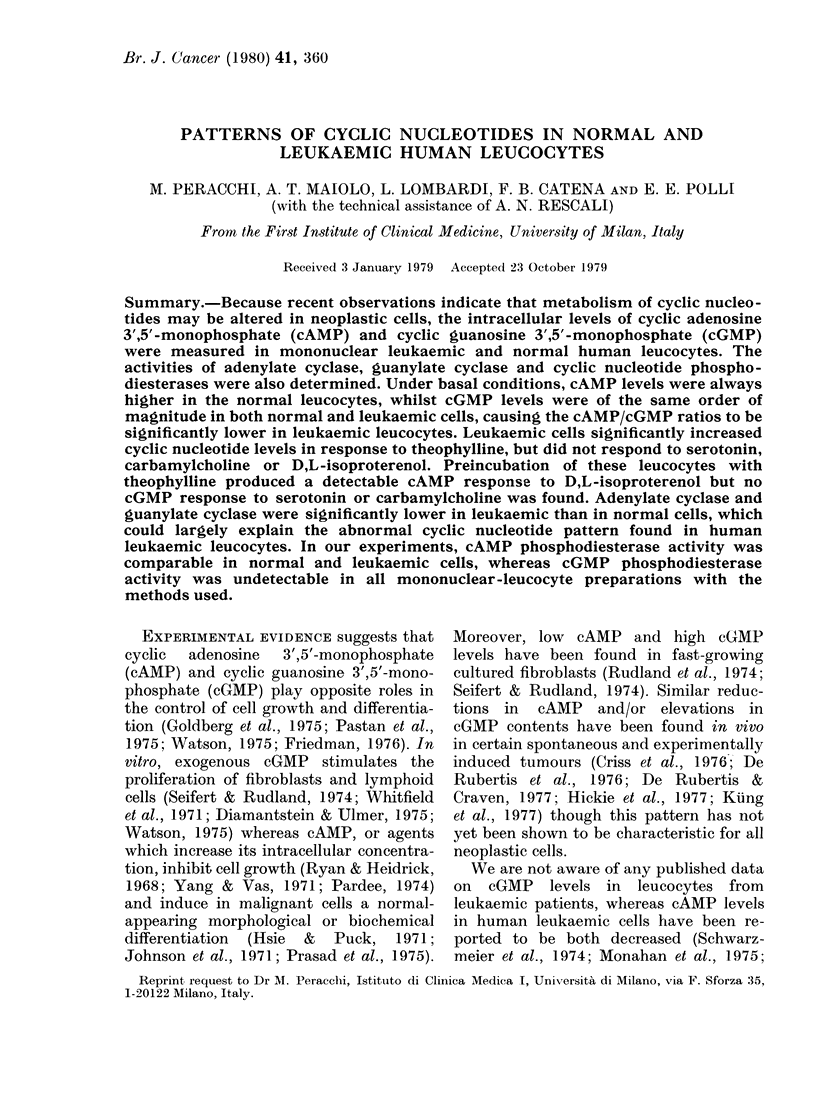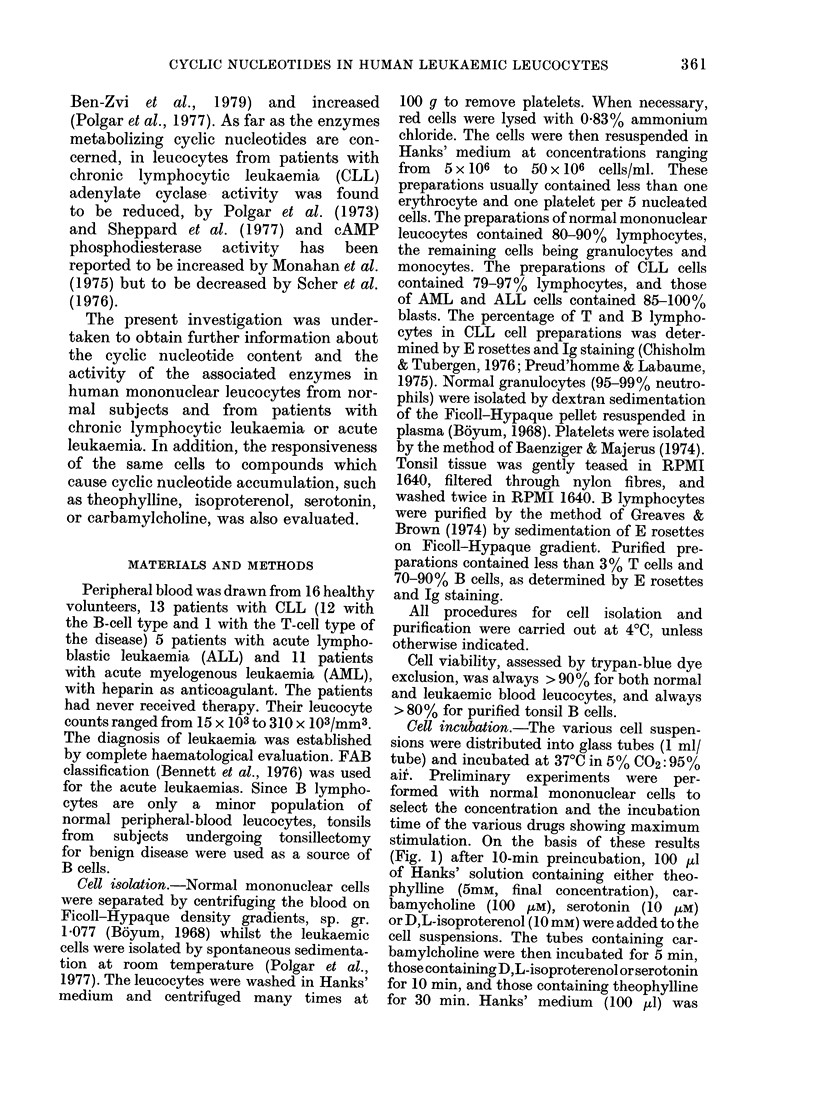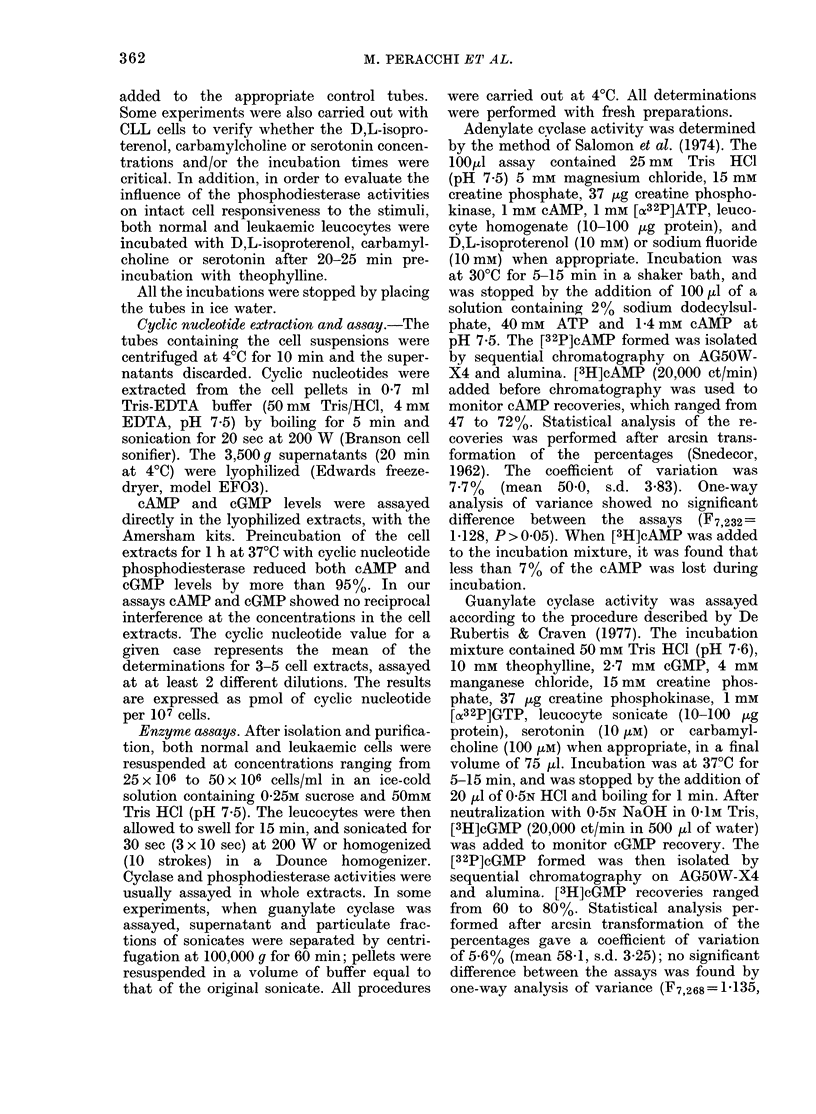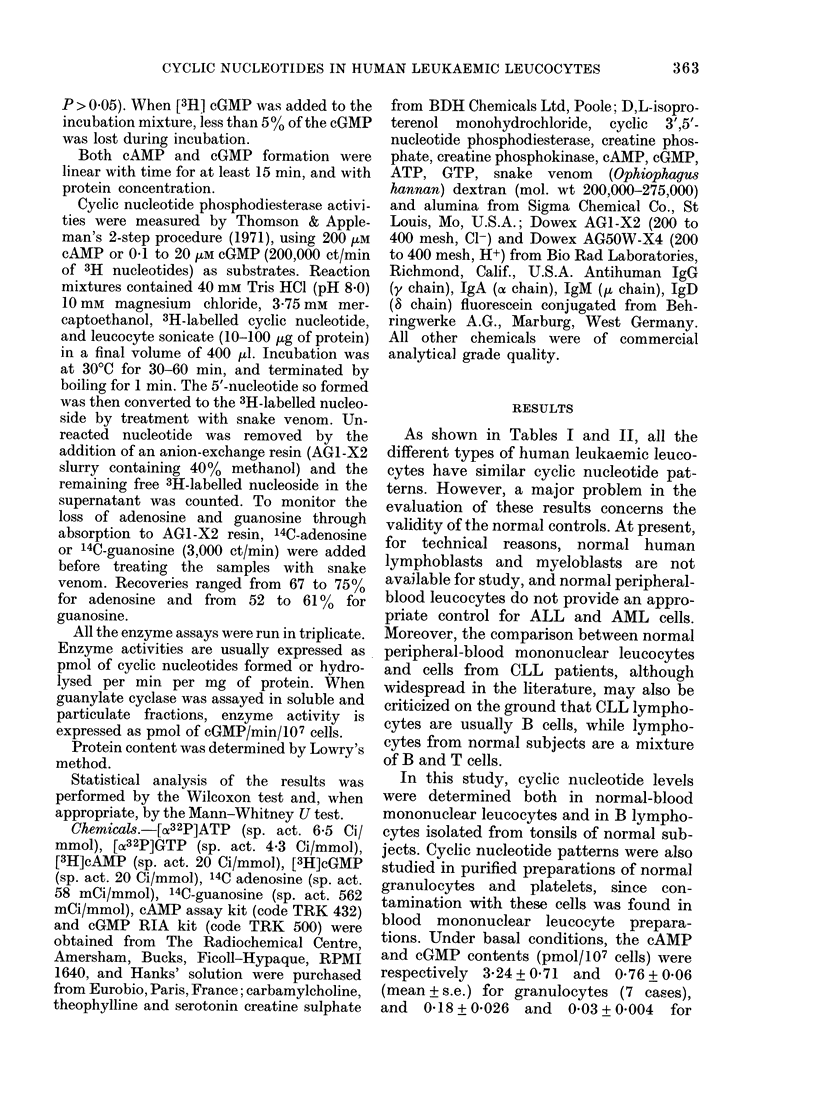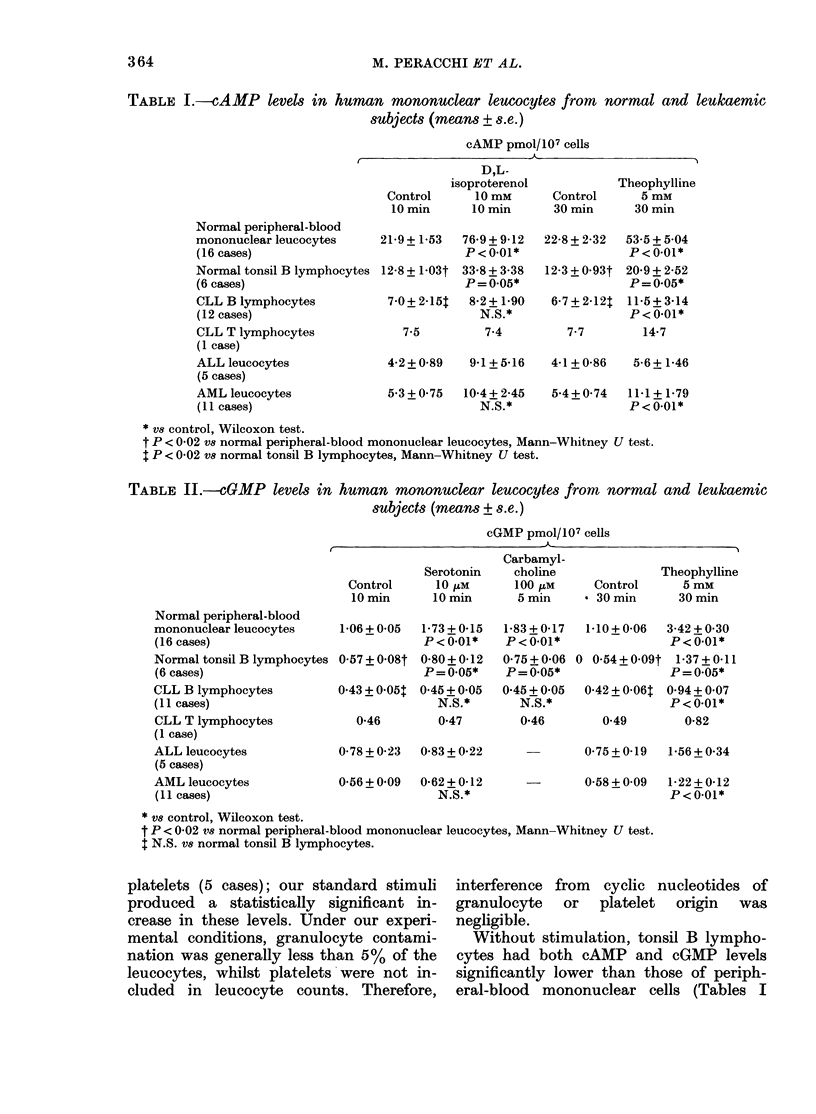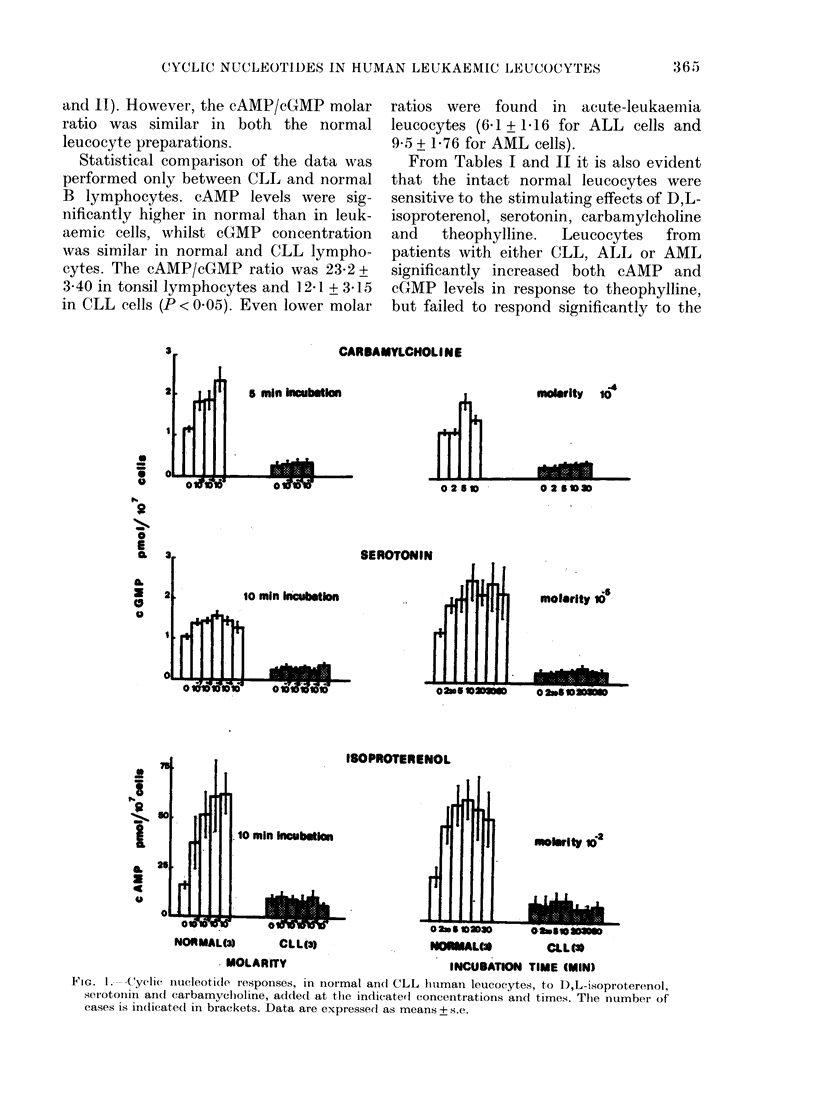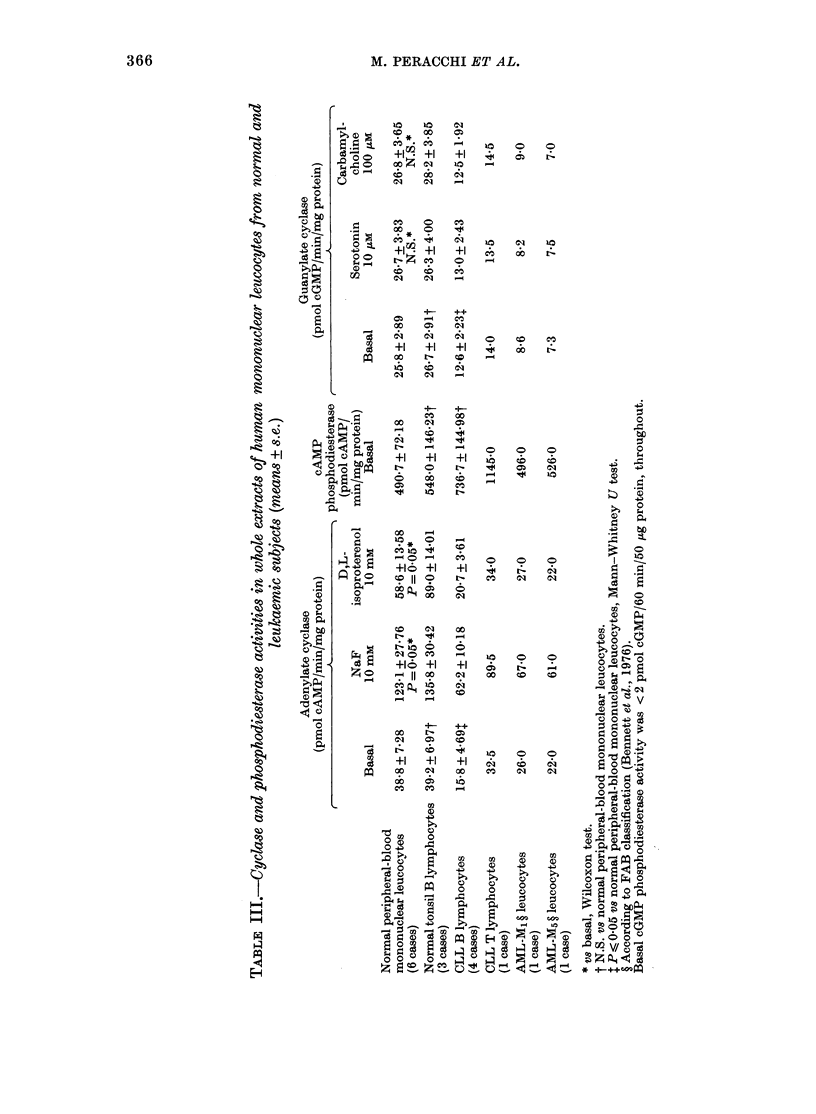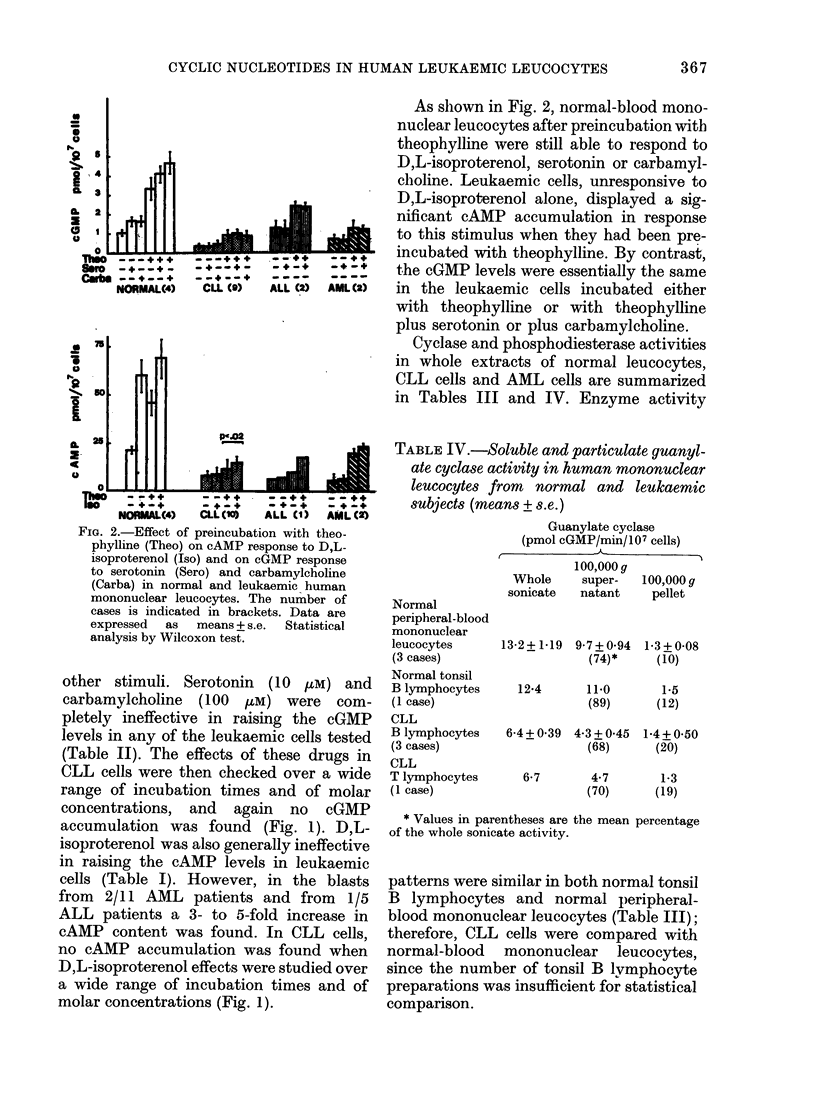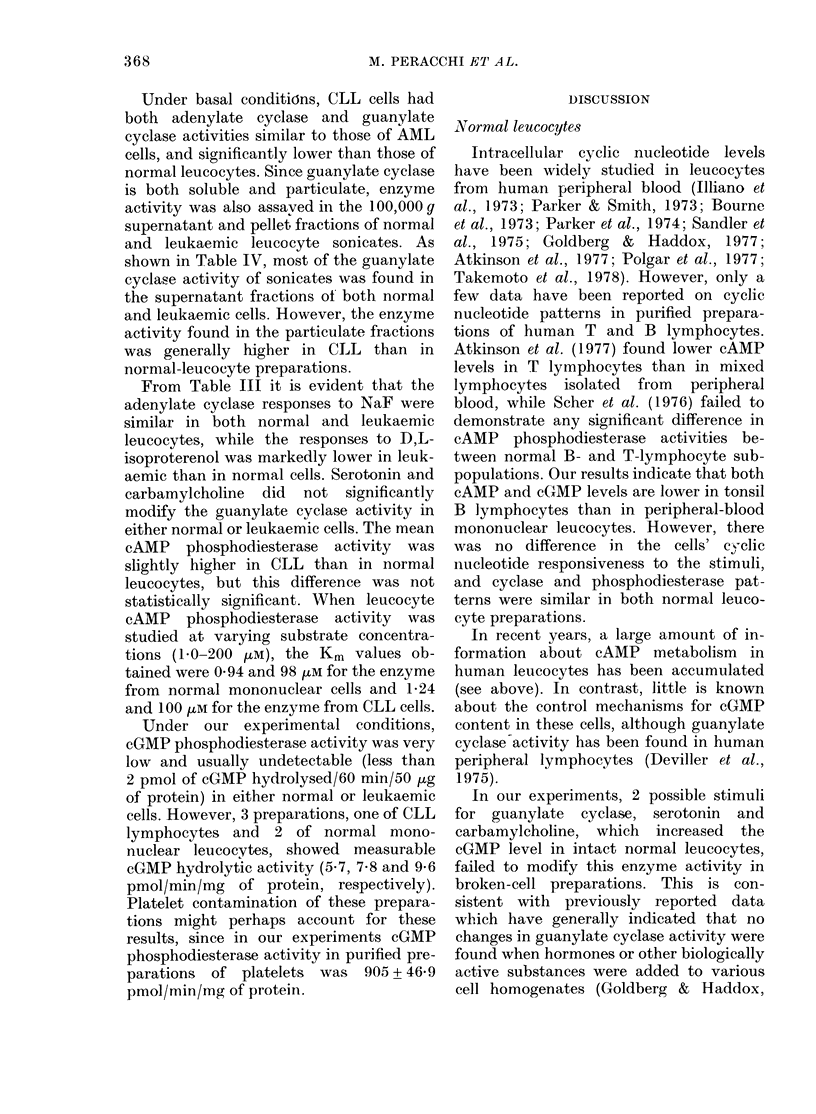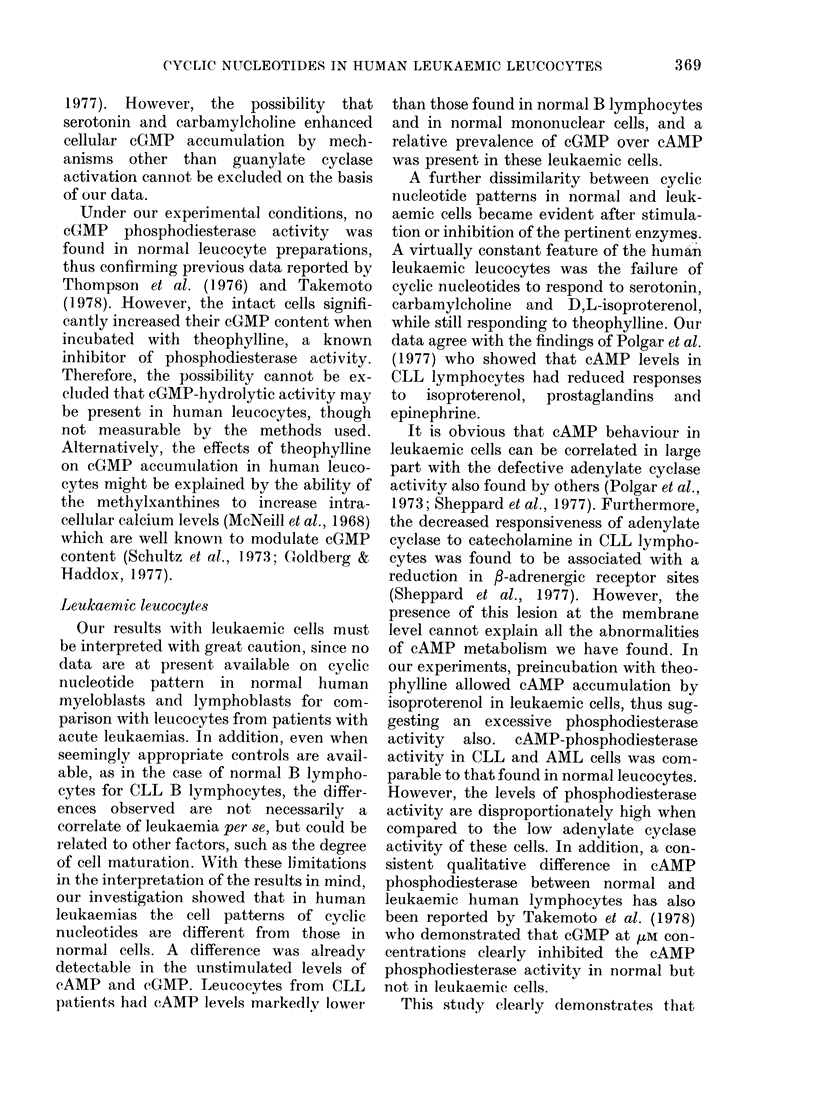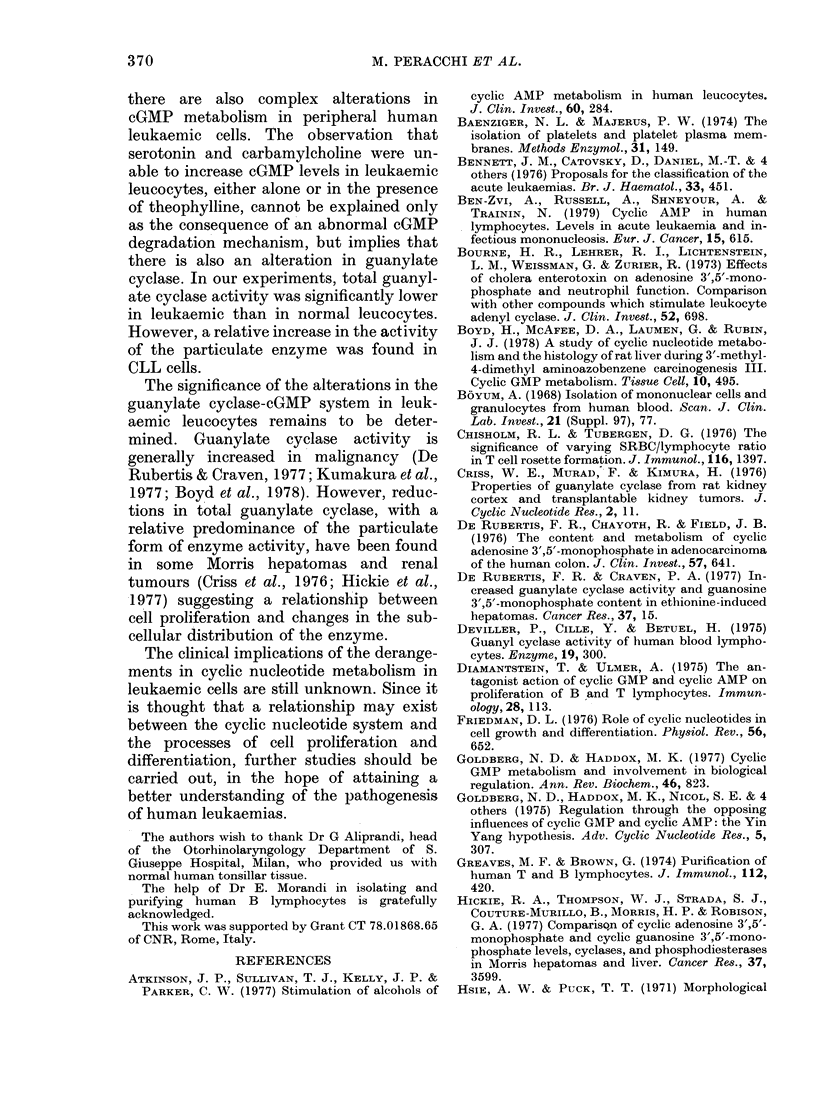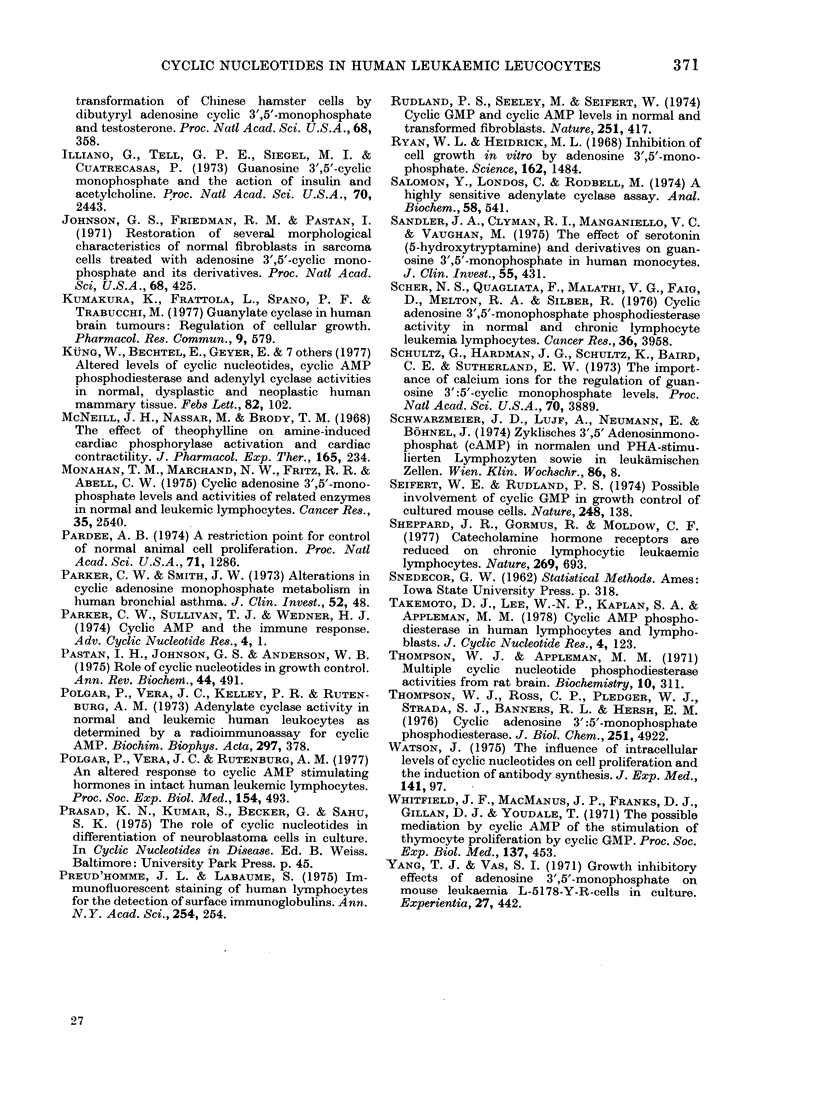# Patterns of cyclic nucleotides in normal and leukaemic human leucocytes.

**DOI:** 10.1038/bjc.1980.59

**Published:** 1980-03

**Authors:** M. Peracchi, A. T. Maiolo, L. Lombardi, F. B. Catena, E. E. Polli

## Abstract

Because recent observations indicate that metabolism of cyclic nucleotides may be altered in neoplastic cells, the intracellular levels of cyclic adenosine 3',5'-monophosphate (cAMP) and cyclic guanosine 3',5'-monophosphate (cGMP) were measured in mononuclear leukaemic and normal human leucocytes. The activities of adenylate cyclase, guanylate cyclase and cyclic nucleotide phosphodiesterases were also determined. Under basal conditions, cAMP levels were always higher in the normal leucocytes, whilst cGMP levels were of the same order of magnitude in both normal and leukaemic cells, causing the cAMP/cGMP ratios to be significantly lower in leukaemic leucocytes. Leukaemic cells significantly increased cyclic nucleotide levels in response to theophylline, but did not respond to serotonin, carbamylcholine or D,L-isoproterenol. Preincubation of these leucocytes with theophylline produced a detectable cAMP response to D,L-isoproterenol but no cGMP response to serotonin or carbamylcholine was found. Adenylate cyclase and guanylate cyclase were significantly lower in leukaemic than in normal cells, which could largely explain the abnormal cyclic nucleotide pattern found in human leukaemic leucocytes. In our experiments, cAMP phosphodiesterase activity was comparable in normal and leukaemic cells, whereas cGMP phosphodiesterase activity was undetectable inall mononuclear-leucocyte preparations with the methods used.


*Br. J. Cancer* (1980) **41**, 360

### PATTERNS OF CYCLIC NUCLEOTIDES IN NORMAL AND LEUKAEMIC HUMAN LEUCOCYTES

M. PERACCHI, A. T. MAIOLO, L. LOMBARDI, F. B. CATENA AND E. E. POLLI
(with the technical assistance of A. N. RESCALI)

*From the First Institute of Clinical Medicine, University of Milan, Italy*

Received 3 January 1979 Accepted 23 October 1979

**Summary.—Because recent observations indicate that metabolism of cyclic nucleotides may be altered in neoplastic cells, the intracellular levels of cyclic adenosine 3′,5′-monophosphate (cAMP) and cyclic guanosine 3′,5′-monophosphate (cGMP) were measured in mononuclear leukaemic and normal human leucocytes. The activities of adenylate cyclase, guanylate cyclase and cyclic nucleotide phosphodiesterases were also determined. Under basal conditions, cAMP levels were always higher in the normal leucocytes, whilst cGMP levels were of the same order of magnitude in both normal and leukaemic cells, causing the cAMP/cGMP ratios to be significantly lower in leukaemic leucocytes. Leukaemic cells significantly increased cyclic nucleotide levels in response to theophylline, but did not respond to serotonin, carbamylcholine or D,L-isoproterenol. Preincubation of these leucocytes with theophylline produced a detectable cAMP response to D,L-isoproterenol but no cGMP response to serotonin or carbamylcholine was found. Adenylate cyclase and guanylate cyclase were significantly lower in leukaemic than in normal cells, which could largely explain the abnormal cyclic nucleotide pattern found in human leukaemic leucocytes. In our experiments, cAMP phosphodiesterase activity was comparable in normal and leukaemic cells, whereas cGMP phosphodiesterase activity was undetectable in all mononuclear-leucocyte preparations with the methods used.**

EXPERIMENTAL EVIDENCE suggests that cyclic adenosine 3′,5′-monophosphate (cAMP) and cyclic guanosine 3′,5′-monophosphate (cGMP) play opposite roles in the control of cell growth and differentiation (Goldberg *et al.*, 1975; Pastan *et al.*, 1975; Watson, 1975; Friedman, 1976). *In vitro*, exogenous cGMP stimulates the proliferation of fibroblasts and lymphoid cells (Seifert & Rudland, 1974; Whitfield *et al.*, 1971; Diamantstein & Ulmer, 1975; Watson, 1975) whereas cAMP, or agents which increase its intracellular concentration, inhibit cell growth (Ryan & Heidrick, 1968; Yang & Vas, 1971; Pardee, 1974) and induce in malignant cells a normal-appearing morphological or biochemical differentiation (Hsie & Puck, 1971; Johnson *et al.*, 1971; Prasad *et al.*, 1975). Moreover, low cAMP and high cGMP levels have been found in fast-growing cultured fibroblasts (Rudland *et al.*, 1974; Seifert & Rudland, 1974). Similar reductions in cAMP and/or elevations in cGMP contents have been found *in vivo* in certain spontaneous and experimentally induced tumours (Criss *et al.*, 1976; De Rubertis *et al.*, 1976; De Rubertis & Craven, 1977; Hickie *et al.*, 1977; Küng *et al.*, 1977) though this pattern has not yet been shown to be characteristic for all neoplastic cells.

We are not aware of any published data on cGMP levels in leucocytes from leukaemic patients, whereas cAMP levels in human leukaemic cells have been reported to be both decreased (Schwarzmeier *et al.*, 1974; Monahan *et al.*, 1975;

Reprint request to Dr M. Peracchi, Istituto di Clinica Medica I, Università di Milano, via F. Sforza 35, I-20122 Milano, Italy.

Ben-Zvi *et al.*, 1979) and increased (Polgar *et al.*, 1977). As far as the enzymes metabolizing cyclic nucleotides are concerned, in leucocytes from patients with chronic lymphocytic leukaemia (CLL) adenylate cyclase activity was found to be reduced, by Polgar *et al.* (1973) and Sheppard *et al.* (1977) and cAMP phosphodiesterase activity has been reported to be increased by Monahan *et al.* (1975) but to be decreased by Scher *et al.* (1976).

The present investigation was undertaken to obtain further information about the cyclic nucleotide content and the activity of the associated enzymes in human mononuclear leucocytes from normal subjects and from patients with chronic lymphocytic leukaemia or acute leukaemia. In addition, the responsiveness of the same cells to compounds which cause cyclic nucleotide accumulation, such as theophylline, isoproterenol, serotonin, or carbamylcholine, was also evaluated.

### MATERIALS AND METHODS

Peripheral blood was drawn from 16 healthy volunteers, 13 patients with CLL (12 with the B-cell type and 1 with the T-cell type of the disease) 5 patients with acute lymphoblastic leukaemia (ALL) and 11 patients with acute myelogenous leukaemia (AML), with heparin as anticoagulant. The patients had never received therapy. Their leucocyte counts ranged from $15 \times 10^3$ to $310 \times 10^3$/mm$^3$. The diagnosis of leukaemia was established by complete haematological evaluation. FAB classification (Bennett *et al.*, 1976) was used for the acute leukaemias. Since B lymphocytes are only a minor population of normal peripheral-blood leucocytes, tonsils from subjects undergoing tonsillectomy for benign disease were used as a source of B cells.

*Cell isolation.*—Normal mononuclear cells were separated by centrifuging the blood on Ficoll–Hypaque density gradients, sp. gr. 1·077 (Böyum, 1968) whilst the leukaemic cells were isolated by spontaneous sedimentation at room temperature (Polgar *et al.*, 1977). The leucocytes were washed in Hanks' medium and centrifuged many times at 100 *g* to remove platelets. When necessary, red cells were lysed with 0·83% ammonium chloride. The cells were then resuspended in Hanks' medium at concentrations ranging from $5 \times 10^6$ to $50 \times 10^6$ cells/ml. These preparations usually contained less than one erythrocyte and one platelet per 5 nucleated cells. The preparations of normal mononuclear leucocytes contained 80–90% lymphocytes, the remaining cells being granulocytes and monocytes. The preparations of CLL cells contained 79–97% lymphocytes, and those of AML and ALL cells contained 85–100% blasts. The percentage of T and B lymphocytes in CLL cell preparations was determined by E rosettes and Ig staining (Chisholm & Tubergen, 1976; Preud'homme & Labaume, 1975). Normal granulocytes (95–99% neutrophils) were isolated by dextran sedimentation of the Ficoll–Hypaque pellet resuspended in plasma (Böyum, 1968). Platelets were isolated by the method of Baenziger & Majerus (1974). Tonsil tissue was gently teased in RPMI 1640, filtered through nylon fibres, and washed twice in RPMI 1640. B lymphocytes were purified by the method of Greaves & Brown (1974) by sedimentation of E rosettes on Ficoll–Hypaque gradient. Purified preparations contained less than 3% T cells and 70–90% B cells, as determined by E rosettes and Ig staining.

All procedures for cell isolation and purification were carried out at 4°C, unless otherwise indicated.

Cell viability, assessed by trypan-blue dye exclusion, was always >90% for both normal and leukaemic blood leucocytes, and always >80% for purified tonsil B cells.

*Cell incubation.*—The various cell suspensions were distributed into glass tubes (1 ml/tube) and incubated at 37°C in 5% $CO_2$:95% air. Preliminary experiments were performed with normal mononuclear cells to select the concentration and the incubation time of the various drugs showing maximum stimulation. On the basis of these results (Fig. 1) after 10-min preincubation, 100 $\mu$l of Hanks' solution containing either theophylline (5mM, final concentration), carbamycholine (100 $\mu$M), serotonin (10 $\mu$M) or D,L-isoproterenol (10 mM) were added to the cell suspensions. The tubes containing carbamylcholine were then incubated for 5 min, those containing D,L-isoproterenol or serotonin for 10 min, and those containing theophylline for 30 min. Hanks' medium (100 $\mu$l) was added to the appropriate control tubes. Some experiments were also carried out with CLL cells to verify whether the D,L-isoproterenol, carbamylcholine or serotonin concentrations and/or the incubation times were critical. In addition, in order to evaluate the influence of the phosphodiesterase activities on intact cell responsiveness to the stimuli, both normal and leukaemic leucocytes were incubated with D,L-isoproterenol, carbamylcholine or serotonin after 20–25 min preincubation with theophylline.

All the incubations were stopped by placing the tubes in ice water.

*Cyclic nucleotide extraction and assay.*—The tubes containing the cell suspensions were centrifuged at 4°C for 10 min and the supernatants discarded. Cyclic nucleotides were extracted from the cell pellets in 0·7 ml Tris-EDTA buffer (50 mM Tris/HCl, 4 mM EDTA, pH 7·5) by boiling for 5 min and sonication for 20 sec at 200 W (Branson cell sonifier). The 3,500 *g* supernatants (20 min at 4°C) were lyophilized (Edwards freeze-dryer, model EFO3).

cAMP and cGMP levels were assayed directly in the lyophilized extracts, with the Amersham kits. Preincubation of the cell extracts for 1 h at 37°C with cyclic nucleotide phosphodiesterase reduced both cAMP and cGMP levels by more than 95%. In our assays cAMP and cGMP showed no reciprocal interference at the concentrations in the cell extracts. The cyclic nucleotide value for a given case represents the mean of the determinations for 3–5 cell extracts, assayed at at least 2 different dilutions. The results are expressed as pmol of cyclic nucleotide per $10^7$ cells.

*Enzyme assays.* After isolation and purification, both normal and leukaemic cells were resuspended at concentrations ranging from $25\times10^6$ to $50\times10^6$ cells/ml in an ice-cold solution containing 0·25M sucrose and 50mM Tris HCl (pH 7·5). The leucocytes were then allowed to swell for 15 min, and sonicated for 30 sec (3 × 10 sec) at 200 W or homogenized (10 strokes) in a Dounce homogenizer. Cyclase and phosphodiesterase activities were usually assayed in whole extracts. In some experiments, when guanylate cyclase was assayed, supernatant and particulate fractions of sonicates were separated by centrifugation at 100,000 *g* for 60 min; pellets were resuspended in a volume of buffer equal to that of the original sonicate. All procedures were carried out at 4°C. All determinations were performed with fresh preparations.

Adenylate cyclase activity was determined by the method of Salomon *et al.* (1974). The 100 μl assay contained 25 mM Tris HCl (pH 7·5) 5 mM magnesium chloride, 15 mM creatine phosphate, 37 μg creatine phosphokinase, 1 mM cAMP, 1 mM [$\alpha^{32}$P]ATP, leucocyte homogenate (10–100 μg protein), and D,L-isoproterenol (10 mM) or sodium fluoride (10 mM) when appropriate. Incubation was at 30°C for 5–15 min in a shaker bath, and was stopped by the addition of 100 μl of a solution containing 2% sodium dodecylsulphate, 40 mM ATP and 1·4 mM cAMP at pH 7·5. The [$^{32}$P]cAMP formed was isolated by sequential chromatography on AG50W-X4 and alumina. [$^{3}$H]cAMP (20,000 ct/min) added before chromatography was used to monitor cAMP recoveries, which ranged from 47 to 72%. Statistical analysis of the recoveries was performed after arcsin transformation of the percentages (Snedecor, 1962). The coefficient of variation was 7·7% (mean 50·0, s.d. 3·83). One-way analysis of variance showed no significant difference between the assays ($F_{7,232}$ = 1·128, $P > 0·05$). When [$^{3}$H]cAMP was added to the incubation mixture, it was found that less than 7% of the cAMP was lost during incubation.

Guanylate cyclase activity was assayed according to the procedure described by De Rubertis & Craven (1977). The incubation mixture contained 50 mM Tris HCl (pH 7·6), 10 mM theophylline, 2·7 mM cGMP, 4 mM manganese chloride, 15 mM creatine phosphate, 37 μg creatine phosphokinase, 1 mM [$\alpha^{32}$P]GTP, leucocyte sonicate (10–100 μg protein), serotonin (10 μM) or carbamylcholine (100 μM) when appropriate, in a final volume of 75 μl. Incubation was at 37°C for 5–15 min, and was stopped by the addition of 20 μl of 0·5N HCl and boiling for 1 min. After neutralization with 0·5N NaOH in 0·1M Tris, [$^{3}$H]cGMP (20,000 ct/min in 500 μl of water) was added to monitor cGMP recovery. The [$^{32}$P]cGMP formed was then isolated by sequential chromatography on AG50W-X4 and alumina. [$^{3}$H]cGMP recoveries ranged from 60 to 80%. Statistical analysis performed after arcsin transformation of the percentages gave a coefficient of variation of 5·6% (mean 58·1, s.d. 3·25); no significant difference between the assays was found by one-way analysis of variance ($F_{7,268}$ = 1·135,

$P > 0.05$). When [$^3$H] cGMP was added to the incubation mixture, less than 5% of the cGMP was lost during incubation.

Both cAMP and cGMP formation were linear with time for at least 15 min, and with protein concentration.

Cyclic nucleotide phosphodiesterase activities were measured by Thomson & Appleman's 2-step procedure (1971), using 200 $\mu$M cAMP or 0·1 to 20 $\mu$M cGMP (200,000 ct/min of $^3$H nucleotides) as substrates. Reaction mixtures contained 40 mM Tris HCl (pH 8·0) 10 mM magnesium chloride, 3·75 mM mercaptoethanol, $^3$H-labelled cyclic nucleotide, and leucocyte sonicate (10–100 $\mu$g of protein) in a final volume of 400 $\mu$l. Incubation was at 30°C for 30–60 min, and terminated by boiling for 1 min. The 5′-nucleotide so formed was then converted to the $^3$H-labelled nucleoside by treatment with snake venom. Unreacted nucleotide was removed by the addition of an anion-exchange resin (AG1-X2 slurry containing 40% methanol) and the remaining free $^3$H-labelled nucleoside in the supernatant was counted. To monitor the loss of adenosine and guanosine through absorption to AG1-X2 resin, $^{14}$C-adenosine or $^{14}$C-guanosine (3,000 ct/min) were added before treating the samples with snake venom. Recoveries ranged from 67 to 75% for adenosine and from 52 to 61% for guanosine.

All the enzyme assays were run in triplicate. Enzyme activities are usually expressed as pmol of cyclic nucleotides formed or hydrolysed per min per mg of protein. When guanylate cyclase was assayed in soluble and particulate fractions, enzyme activity is expressed as pmol of cGMP/min/$10^7$ cells.

Protein content was determined by Lowry's method.

Statistical analysis of the results was performed by the Wilcoxon test and, when appropriate, by the Mann–Whitney $U$ test.

*Chemicals.*—[$\alpha^{32}$P]ATP (sp. act. 6·5 Ci/mmol), [$\alpha^{32}$P]GTP (sp. act. 4·3 Ci/mmol), [$^3$H]cAMP (sp. act. 20 Ci/mmol), [$^3$H]cGMP (sp. act. 20 Ci/mmol), $^{14}$C adenosine (sp. act. 58 mCi/mmol), $^{14}$C-guanosine (sp. act. 562 mCi/mmol), cAMP assay kit (code TRK 432) and cGMP RIA kit (code TRK 500) were obtained from The Radiochemical Centre, Amersham, Bucks, Ficoll–Hypaque, RPMI 1640, and Hanks' solution were purchased from Eurobio, Paris, France; carbamylcholine, theophylline and serotonin creatine sulphate from BDH Chemicals Ltd, Poole; D,L-isoproterenol monohydrochloride, cyclic 3′,5′-nucleotide phosphodiesterase, creatine phosphate, creatine phosphokinase, cAMP, cGMP, ATP, GTP, snake venom (*Ophiophagus hannan*) dextran (mol. wt 200,000–275,000) and alumina from Sigma Chemical Co., St Louis, Mo, U.S.A.; Dowex AG1-X2 (200 to 400 mesh, $Cl^-$) and Dowex AG50W-X4 (200 to 400 mesh, $H^+$) from Bio Rad Laboratories, Richmond, Calif., U.S.A. Antihuman IgG ($\gamma$ chain), IgA ($\alpha$ chain), IgM ($\mu$ chain), IgD ($\delta$ chain) fluorescein conjugated from Behringwerke A.G., Marburg, West Germany. All other chemicals were of commercial analytical grade quality.

### RESULTS

As shown in Tables I and II, all the different types of human leukaemic leucocytes have similar cyclic nucleotide patterns. However, a major problem in the evaluation of these results concerns the validity of the normal controls. At present, for technical reasons, normal human lymphoblasts and myeloblasts are not available for study, and normal peripheral-blood leucocytes do not provide an appropriate control for ALL and AML cells. Moreover, the comparison between normal peripheral-blood mononuclear leucocytes and cells from CLL patients, although widespread in the literature, may also be criticized on the ground that CLL lymphocytes are usually B cells, while lymphocytes from normal subjects are a mixture of B and T cells.

In this study, cyclic nucleotide levels were determined both in normal-blood mononuclear leucocytes and in B lymphocytes isolated from tonsils of normal subjects. Cyclic nucleotide patterns were also studied in purified preparations of normal granulocytes and platelets, since contamination with these cells was found in blood mononuclear leucocyte preparations. Under basal conditions, the cAMP and cGMP contents (pmol/$10^7$ cells) were respectively $3.24 \pm 0.71$ and $0.76 \pm 0.06$ (mean ± s.e.) for granulocytes (7 cases), and $0.18 \pm 0.026$ and $0.03 \pm 0.004$ for

TABLE I.—*cAMP levels in human mononuclear leucocytes from normal and leukaemic subjects (means ± s.e.)*

| | cAMP pmol/$10^7$ cells | | | |
|---|---|---|---|---|
| | Control 10 min | D,L-isoproterenol 10 mM 10 min | Control 30 min | Theophylline 5 mM 30 min |
| Normal peripheral-blood mononuclear leucocytes (16 cases) | 21·9 ± 1·53 | 76·9 ± 9·12 $P < 0·01$* | 22·8 ± 2·32 | 53·5 ± 5·04 $P < 0·01$* |
| Normal tonsil B lymphocytes (6 cases) | 12·8 ± 1·03† | 33·8 ± 3·38 $P = 0·05$* | 12·3 ± 0·93† | 20·9 ± 2·52 $P = 0·05$* |
| CLL B lymphocytes (12 cases) | 7·0 ± 2·15‡ | 8·2 ± 1·90 N.S.* | 6·7 ± 2·12‡ | 11·5 ± 3·14 $P < 0·01$* |
| CLL T lymphocytes (1 case) | 7·5 | 7·4 | 7·7 | 14·7 |
| ALL leucocytes (5 cases) | 4·2 ± 0·89 | 9·1 ± 5·16 | 4·1 ± 0·86 | 5·6 ± 1·46 |
| AML leucocytes (11 cases) | 5·3 ± 0·75 | 10·4 ± 2·45 N.S.* | 5·4 ± 0·74 | 11·1 ± 1·79 $P < 0·01$* |

* *vs* control, Wilcoxon test.
† $P < 0·02$ *vs* normal peripheral-blood mononuclear leucocytes, Mann–Whitney $U$ test.
‡ $P < 0·02$ *vs* normal tonsil B lymphocytes, Mann–Whitney $U$ test.

TABLE II.—*cGMP levels in human mononuclear leucocytes from normal and leukaemic subjects (means ± s.e.)*

| | cGMP pmol/$10^7$ cells | | | | |
|---|---|---|---|---|---|
| | Control 10 min | Serotonin 10 μM 10 min | Carbamyl-choline 100 μM 5 min | Control 30 min | Theophylline 5 mM 30 min |
| Normal peripheral-blood mononuclear leucocytes (16 cases) | 1·06 ± 0·05 | 1·73 ± 0·15 $P < 0·01$* | 1·83 ± 0·17 $P < 0·01$* | 1·10 ± 0·06 | 3·42 ± 0·30 $P < 0·01$* |
| Normal tonsil B lymphocytes (6 cases) | 0·57 ± 0·08† | 0·80 ± 0·12 $P = 0·05$* | 0·75 ± 0·06 $P = 0·05$* | 0 0·54 ± 0·09† | 1·37 ± 0·11 $P = 0·05$* |
| CLL B lymphocytes (11 cases) | 0·43 ± 0·05‡ | 0·45 ± 0·05 N.S.* | 0·45 ± 0·05 N.S.* | 0·42 ± 0·06‡ | 0·94 ± 0·07 $P < 0·01$* |
| CLL T lymphocytes (1 case) | 0·46 | 0·47 | 0·46 | 0·49 | 0·82 |
| ALL leucocytes (5 cases) | 0·78 ± 0·23 | 0·83 ± 0·22 | — | 0·75 ± 0·19 | 1·56 ± 0·34 |
| AML leucocytes (11 cases) | 0·56 ± 0·09 | 0·62 ± 0·12 N.S.* | — | 0·58 ± 0·09 | 1·22 ± 0·12 $P < 0·01$* |

* *vs* control, Wilcoxon test.
† $P < 0·02$ *vs* normal peripheral-blood mononuclear leucocytes, Mann–Whitney $U$ test.
‡ N.S. *vs* normal tonsil B lymphocytes.

platelets (5 cases); our standard stimuli produced a statistically significant increase in these levels. Under our experimental conditions, granulocyte contamination was generally less than 5% of the leucocytes, whilst platelets were not included in leucocyte counts. Therefore, interference from cyclic nucleotides of granulocyte or platelet origin was negligible.

Without stimulation, tonsil B lymphocytes had both cAMP and cGMP levels significantly lower than those of peripheral-blood mononuclear cells (Tables I

and II). However, the cAMP/cGMP molar ratio was similar in both the normal leucocyte preparations.

Statistical comparison of the data was performed only between CLL and normal B lymphocytes. cAMP levels were significantly higher in normal than in leukaemic cells, whilst cGMP concentration was similar in normal and CLL lymphocytes. The cAMP/cGMP ratio was $23 \cdot 2 \pm 3 \cdot 40$ in tonsil lymphocytes and $12 \cdot 1 \pm 3 \cdot 15$ in CLL cells ($P < 0 \cdot 05$). Even lower molar ratios were found in acute-leukaemia leucocytes ($6 \cdot 1 \pm 1 \cdot 16$ for ALL cells and $9 \cdot 5 \pm 1 \cdot 76$ for AML cells).

From Tables I and II it is also evident that the intact normal leucocytes were sensitive to the stimulating effects of D,L-isoproterenol, serotonin, carbamylcholine and theophylline. Leucocytes from patients with either CLL, ALL or AML significantly increased both cAMP and cGMP levels in response to theophylline, but failed to respond significantly to the

FIG. 1.—Cyclic nucleotide responses, in normal and CLL human leucocytes, to D,L-isoproterenol, serotonin and carbamycholine, added at the indicated concentrations and times. The number of cases is indicated in brackets. Data are expressed as means ± s.e.

TABLE III.—*Cyclase and phosphodiesterase activities in whole extracts of human mononuclear leucocytes from normal and leukaemic subjects (means ± s.e.)*

| | Adenylate cyclase (pmol cAMP/min/mg protein) | | | cAMP phosphodiesterase (pmol cAMP/min/mg protein) | Guanylate cyclase (pmol cGMP/min/mg protein) | | |
|---|---|---|---|---|---|---|---|
| | Basal | NaF 10 mM | D,L-isoproterenol 10 mM | Basal | Basal | Serotonin 10 μM | Carbamylcholine 100 μM |
| Normal peripheral-blood mononuclear leucocytes (6 cases) | 38·8 ± 7·28 | 123·1 ± 27·76 $P = 0{\cdot}05$* | 58·6 ± 13·58 $P = 0{\cdot}05$* | 490·7 ± 72·18 | 25·8 ± 2·89 | 26·7 ± 3·83 N.S.* | 26·8 ± 3·65 N.S.* |
| Normal tonsil B lymphocytes (3 cases) | 39·2 ± 6·97† | 135·8 ± 30·42 | 89·0 ± 14·01 | 548·0 ± 146·23† | 26·7 ± 2·91† | 26·3 ± 4·00 | 28·2 ± 3·85 |
| CLL B lymphocytes (4 cases) | 15·8 ± 4·69‡ | 62·2 ± 10·18 | 20·7 ± 3·61 | 736·7 ± 144·98† | 12·6 ± 2·23‡ | 13·0 ± 2·43 | 12·5 ± 1·92 |
| CLL T lymphocytes (1 case) | 32·5 | 89·5 | 34·0 | 1145·0 | 14·0 | 13·5 | 14·5 |
| AML-$M_1$§ leucocytes (1 case) | 26·0 | 67·0 | 27·0 | 496·0 | 8·6 | 8·2 | 9·0 |
| AML-$M_5$§ leucocytes (1 case) | 22·0 | 61·0 | 22·0 | 526·0 | 7·3 | 7·5 | 7·0 |

* *vs* basal, Wilcoxon test.

† N.S. *vs* normal peripheral-blood mononuclear leucocytes.

‡ $P \leqslant 0{\cdot}05$ *vs* normal peripheral-blood mononuclear leucocytes, Mann–Whitney $U$ test.

§ According to FAB classification (Bennett *et al.*, 1976).

Basal cGMP phosphodiesterase activity was < 2 pmol cGMP/60 min/50 μg protein, throughout.

FIG. 2.—Effect of preincubation with theophylline (Theo) on cAMP response to D,L-isoproterenol (Iso) and on cGMP response to serotonin (Sero) and carbamylcholine (Carba) in normal and leukaemic human mononuclear leucocytes. The number of cases is indicated in brackets. Data are expressed as means ± s.e. Statistical analysis by Wilcoxon test.

other stimuli. Serotonin (10 $\mu$M) and carbamylcholine (100 $\mu$M) were completely ineffective in raising the cGMP levels in any of the leukaemic cells tested (Table II). The effects of these drugs in CLL cells were then checked over a wide range of incubation times and of molar concentrations, and again no cGMP accumulation was found (Fig. 1). D,L-isoproterenol was also generally ineffective in raising the cAMP levels in leukaemic cells (Table I). However, in the blasts from 2/11 AML patients and from 1/5 ALL patients a 3- to 5-fold increase in cAMP content was found. In CLL cells, no cAMP accumulation was found when D,L-isoproterenol effects were studied over a wide range of incubation times and of molar concentrations (Fig. 1).

As shown in Fig. 2, normal-blood mononuclear leucocytes after preincubation with theophylline were still able to respond to D,L-isoproterenol, serotonin or carbamylcholine. Leukaemic cells, unresponsive to D,L-isoproterenol alone, displayed a significant cAMP accumulation in response to this stimulus when they had been preincubated with theophylline. By contrast, the cGMP levels were essentially the same in the leukaemic cells incubated either with theophylline or with theophylline plus serotonin or plus carbamylcholine.

Cyclase and phosphodiesterase activities in whole extracts of normal leucocytes, CLL cells and AML cells are summarized in Tables III and IV. Enzyme activity

TABLE IV.—*Soluble and particulate guanylate cyclase activity in human mononuclear leucocytes from normal and leukaemic subjects (means ± s.e.)*

| | Guanylate cyclase (pmol cGMP/min/$10^7$ cells) | | |
|---|---|---|---|
| | Whole sonicate | 100,000 *g* super-natant | 100,000 *g* pellet |
| Normal peripheral-blood mononuclear leucocytes (3 cases) | 13·2 ± 1·19 | 9·7 ± 0·94 (74)* | 1·3 ± 0·08 (10) |
| Normal tonsil B lymphocytes (1 case) | 12·4 | 11·0 (89) | 1·5 (12) |
| CLL B lymphocytes (3 cases) | 6·4 ± 0·39 | 4·3 ± 0·45 (68) | 1·4 ± 0·50 (20) |
| CLL T lymphocytes (1 case) | 6·7 | 4·7 (70) | 1·3 (19) |

* Values in parentheses are the mean percentage of the whole sonicate activity.

patterns were similar in both normal tonsil B lymphocytes and normal peripheral-blood mononuclear leucocytes (Table III); therefore, CLL cells were compared with normal-blood mononuclear leucocytes, since the number of tonsil B lymphocyte preparations was insufficient for statistical comparison.

Under basal conditions, CLL cells had both adenylate cyclase and guanylate cyclase activities similar to those of AML cells, and significantly lower than those of normal leucocytes. Since guanylate cyclase is both soluble and particulate, enzyme activity was also assayed in the 100,000 *g* supernatant and pellet fractions of normal and leukaemic leucocyte sonicates. As shown in Table IV, most of the guanylate cyclase activity of sonicates was found in the supernatant fractions of both normal and leukaemic cells. However, the enzyme activity found in the particulate fractions was generally higher in CLL than in normal-leucocyte preparations.

From Table III it is evident that the adenylate cyclase responses to NaF were similar in both normal and leukaemic leucocytes, while the responses to D,L-isoproterenol was markedly lower in leukaemic than in normal cells. Serotonin and carbamylcholine did not significantly modify the guanylate cyclase activity in either normal or leukaemic cells. The mean cAMP phosphodiesterase activity was slightly higher in CLL than in normal leucocytes, but this difference was not statistically significant. When leucocyte cAMP phosphodiesterase activity was studied at varying substrate concentrations (1·0–200 $\mu$M), the $K_m$ values obtained were 0·94 and 98 $\mu$M for the enzyme from normal mononuclear cells and 1·24 and 100 $\mu$M for the enzyme from CLL cells.

Under our experimental conditions, cGMP phosphodiesterase activity was very low and usually undetectable (less than 2 pmol of cGMP hydrolysed/60 min/50 $\mu$g of protein) in either normal or leukaemic cells. However, 3 preparations, one of CLL lymphocytes and 2 of normal mononuclear leucocytes, showed measurable cGMP hydrolytic activity (5·7, 7·8 and 9·6 pmol/min/mg of protein, respectively). Platelet contamination of these preparations might perhaps account for these results, since in our experiments cGMP phosphodiesterase activity in purified preparations of platelets was $905 \pm 46{\cdot}9$ pmol/min/mg of protein.

### DISCUSSION

### *Normal leucocytes*

Intracellular cyclic nucleotide levels have been widely studied in leucocytes from human peripheral blood (Illiano *et al.*, 1973; Parker & Smith, 1973; Bourne *et al.*, 1973; Parker *et al.*, 1974; Sandler *et al.*, 1975; Goldberg & Haddox, 1977; Atkinson *et al.*, 1977; Polgar *et al.*, 1977; Takemoto *et al.*, 1978). However, only a few data have been reported on cyclic nucleotide patterns in purified preparations of human T and B lymphocytes. Atkinson *et al.* (1977) found lower cAMP levels in T lymphocytes than in mixed lymphocytes isolated from peripheral blood, while Scher *et al.* (1976) failed to demonstrate any significant difference in cAMP phosphodiesterase activities between normal B- and T-lymphocyte subpopulations. Our results indicate that both cAMP and cGMP levels are lower in tonsil B lymphocytes than in peripheral-blood mononuclear leucocytes. However, there was no difference in the cells' cyclic nucleotide responsiveness to the stimuli, and cyclase and phosphodiesterase patterns were similar in both normal leucocyte preparations.

In recent years, a large amount of information about cAMP metabolism in human leucocytes has been accumulated (see above). In contrast, little is known about the control mechanisms for cGMP content in these cells, although guanylate cyclase activity has been found in human peripheral lymphocytes (Deviller *et al.*, 1975).

In our experiments, 2 possible stimuli for guanylate cyclase, serotonin and carbamylcholine, which increased the cGMP level in intact normal leucocytes, failed to modify this enzyme activity in broken-cell preparations. This is consistent with previously reported data which have generally indicated that no changes in guanylate cyclase activity were found when hormones or other biologically active substances were added to various cell homogenates (Goldberg & Haddox,

1977). However, the possibility that serotonin and carbamylcholine enhanced cellular cGMP accumulation by mechanisms other than guanylate cyclase activation cannot be excluded on the basis of our data.

Under our experimental conditions, no cGMP phosphodiesterase activity was found in normal leucocyte preparations, thus confirming previous data reported by Thompson *et al.* (1976) and Takemoto (1978). However, the intact cells significantly increased their cGMP content when incubated with theophylline, a known inhibitor of phosphodiesterase activity. Therefore, the possibility cannot be excluded that cGMP-hydrolytic activity may be present in human leucocytes, though not measurable by the methods used. Alternatively, the effects of theophylline on cGMP accumulation in human leucocytes might be explained by the ability of the methylxanthines to increase intracellular calcium levels (McNeill *et al.*, 1968) which are well known to modulate cGMP content (Schultz *et al.*, 1973; Goldberg & Haddox, 1977).

*Leukaemic leucocytes*

Our results with leukaemic cells must be interpreted with great caution, since no data are at present available on cyclic nucleotide pattern in normal human myeloblasts and lymphoblasts for comparison with leucocytes from patients with acute leukaemias. In addition, even when seemingly appropriate controls are available, as in the case of normal B lymphocytes for CLL B lymphocytes, the differences observed are not necessarily a correlate of leukaemia *per se*, but could be related to other factors, such as the degree of cell maturation. With these limitations in the interpretation of the results in mind, our investigation showed that in human leukaemias the cell patterns of cyclic nucleotides are different from those in normal cells. A difference was already detectable in the unstimulated levels of cAMP and cGMP. Leucocytes from CLL patients had cAMP levels markedly lower than those found in normal B lymphocytes and in normal mononuclear cells, and a relative prevalence of cGMP over cAMP was present in these leukaemic cells.

A further dissimilarity between cyclic nucleotide patterns in normal and leukaemic cells became evident after stimulation or inhibition of the pertinent enzymes. A virtually constant feature of the human leukaemic leucocytes was the failure of cyclic nucleotides to respond to serotonin, carbamylcholine and D,L-isoproterenol, while still responding to theophylline. Our data agree with the findings of Polgar *et al.* (1977) who showed that cAMP levels in CLL lymphocytes had reduced responses to isoproterenol, prostaglandins and epinephrine.

It is obvious that cAMP behaviour in leukaemic cells can be correlated in large part with the defective adenylate cyclase activity also found by others (Polgar *et al.*, 1973; Sheppard *et al.*, 1977). Furthermore, the decreased responsiveness of adenylate cyclase to catecholamine in CLL lymphocytes was found to be associated with a reduction in $\beta$-adrenergic receptor sites (Sheppard *et al.*, 1977). However, the presence of this lesion at the membrane level cannot explain all the abnormalities of cAMP metabolism we have found. In our experiments, preincubation with theophylline allowed cAMP accumulation by isoproterenol in leukaemic cells, thus suggesting an excessive phosphodiesterase activity also. cAMP-phosphodiesterase activity in CLL and AML cells was comparable to that found in normal leucocytes. However, the levels of phosphodiesterase activity are disproportionately high when compared to the low adenylate cyclase activity of these cells. In addition, a consistent qualitative difference in cAMP phosphodiesterase between normal and leukaemic human lymphocytes has also been reported by Takemoto *et al.* (1978) who demonstrated that cGMP at $\mu$M concentrations clearly inhibited the cAMP phosphodiesterase activity in normal but not in leukaemic cells.

This study clearly demonstrates that there are also complex alterations in cGMP metabolism in peripheral human leukaemic cells. The observation that serotonin and carbamylcholine were unable to increase cGMP levels in leukaemic leucocytes, either alone or in the presence of theophylline, cannot be explained only as the consequence of an abnormal cGMP degradation mechanism, but implies that there is also an alteration in guanylate cyclase. In our experiments, total guanylate cyclase activity was significantly lower in leukaemic than in normal leucocytes. However, a relative increase in the activity of the particulate enzyme was found in CLL cells.

The significance of the alterations in the guanylate cyclase-cGMP system in leukaemic leucocytes remains to be determined. Guanylate cyclase activity is generally increased in malignancy (De Rubertis & Craven, 1977; Kumakura *et al.*, 1977; Boyd *et al.*, 1978). However, reductions in total guanylate cyclase, with a relative predominance of the particulate form of enzyme activity, have been found in some Morris hepatomas and renal tumours (Criss *et al.*, 1976; Hickie *et al.*, 1977) suggesting a relationship between cell proliferation and changes in the subcellular distribution of the enzyme.

The clinical implications of the derangements in cyclic nucleotide metabolism in leukaemic cells are still unknown. Since it is thought that a relationship may exist between the cyclic nucleotide system and the processes of cell proliferation and differentiation, further studies should be carried out, in the hope of attaining a better understanding of the pathogenesis of human leukaemias.

The authors wish to thank Dr G Aliprandi, head of the Otorhinolaryngology Department of S. Giuseppe Hospital, Milan, who provided us with normal human tonsillar tissue.

The help of Dr E. Morandi in isolating and purifying human B lymphocytes is gratefully acknowledged.

This work was supported by Grant CT 78.01868.65 of CNR, Rome, Italy.

### REFERENCES

ATKINSON, J. P., SULLIVAN, T. J., KELLY, J. P. & PARKER, C. W. (1977) Stimulation of alcohols of cyclic AMP metabolism in human leucocytes. *J. Clin. Invest.*, **60**, 284.

BAENZIGER, N. L. & MAJERUS, P. W. (1974) The isolation of platelets and platelet plasma membranes. *Methods Enzymol.*, **31**, 149.

BENNETT, J. M., CATOVSKY, D., DANIEL, M.-T. & 4 others (1976) Proposals for the classification of the acute leukaemias. *Br. J. Haematol.*, **33**, 451.

BEN-ZVI, A., RUSSELL, A., SHNEYOUR, A. & TRAININ, N. (1979) Cyclic AMP in human lymphocytes. Levels in acute leukaemia and infectious mononucleosis. *Eur. J. Cancer*, **15**, 615.

BOURNE, H. R., LEHRER, R. I., LICHTENSTEIN, L. M., WEISSMAN, G. & ZURIER, R. (1973) Effects of cholera enterotoxin on adenosine 3′,5′-monophosphate and neutrophil function. Comparison with other compounds which stimulate leukocyte adenyl cyclase. *J. Clin. Invest.*, **52**, 698.

BOYD, H., MCAFEE, D. A., LAUMEN, G. & RUBIN, J. J. (1978) A study of cyclic nucleotide metabolism and the histology of rat liver during 3′-methyl-4-dimethyl aminoazobenzene carcinogenesis III. Cyclic GMP metabolism. *Tissue Cell*, **10**, 495.

BÖYUM, A. (1968) Isolation of mononuclear cells and granulocytes from human blood. *Scan. J. Clin. Lab. Invest.*, **21** (Suppl. 97), 77.

CHISHOLM, R. L. & TUBERGEN, D. G. (1976) The significance of varying SRBC/lymphocyte ratio in T cell rosette formation. *J. Immunol.*, **116**, 1397.

CRISS, W. E., MURAD, F. & KIMURA, H. (1976) Properties of guanylate cyclase from rat kidney cortex and transplantable kidney tumors. *J. Cyclic Nucleotide Res.*, **2**, 11.

DE RUBERTIS, F. R., CHAYOTH, R. & FIELD, J. B. (1976) The content and metabolism of cyclic adenosine 3′,5′-monophosphate in adenocarcinoma of the human colon. *J. Clin. Invest.*, **57**, 641.

DE RUBERTIS, F. R. & CRAVEN, P. A. (1977) Increased guanylate cyclase activity and guanosine 3′,5′-monophosphate content in ethionine-induced hepatomas. *Cancer Res.*, **37**, 15.

DEVILLER, P., CILLE, Y. & BETUEL, H. (1975) Guanyl cyclase activity of human blood lymphocytes. *Enzyme*, **19**, 300.

DIAMANTSTEIN, T. & ULMER, A. (1975) The antagonist action of cyclic GMP and cyclic AMP on proliferation of B and T lymphocytes. *Immunology*, **28**, 113.

FRIEDMAN, D. L. (1976) Role of cyclic nucleotides in cell growth and differentiation. *Physiol. Rev.*, **56**, 652.

GOLDBERG, N. D. & HADDOX, M. K. (1977) Cyclic GMP metabolism and involvement in biological regulation. *Ann. Rev. Biochem.*, **46**, 823.

GOLDBERG, N. D., HADDOX, M. K., NICOL, S. E. & 4 others (1975) Regulation through the opposing influences of cyclic GMP and cyclic AMP: the Yin Yang hypothesis. *Adv. Cyclic Nucleotide Res.*, **5**, 307.

GREAVES, M. F. & BROWN, G. (1974) Purification of human T and B lymphocytes. *J. Immunol.*, **112**, 420.

HICKIE, R. A., THOMPSON, W. J., STRADA, S. J., COUTURE-MURILLO, B., MORRIS, H. P. & ROBISON, G. A. (1977) Comparison of cyclic adenosine 3′,5′-monophosphate and cyclic guanosine 3′,5′-monophosphate levels, cyclases, and phosphodiesterases in Morris hepatomas and liver. *Cancer Res.*, **37**, 3599.

HSIE, A. W. & PUCK, T. T. (1971) Morphological transformation of Chinese hamster cells by dibutyryl adenosine cyclic 3′,5′-monophosphate and testosterone. *Proc. Natl Acad. Sci. U.S.A.*, **68**, 358.

ILLIANO, G., TELL, G. P. E., SIEGEL, M. I. & CUATRECASAS, P. (1973) Guanosine 3′,5′-cyclic monophosphate and the action of insulin and acetylcholine. *Proc. Natl Acad. Sci. U.S.A.*, **70**, 2443.

JOHNSON, G. S., FRIEDMAN, R. M. & PASTAN, I. (1971) Restoration of several morphological characteristics of normal fibroblasts in sarcoma cells treated with adenosine 3′,5′-cyclic monophosphate and its derivatives. *Proc. Natl Acad. Sci, U.S.A.*, **68**, 425.

KUMAKURA, K., FRATTOLA, L., SPANO, P. F. & TRABUCCHI, M. (1977) Guanylate cyclase in human brain tumours: Regulation of cellular growth. *Pharmacol. Res. Commun.*, **9**, 579.

KÜNG, W., BECHTEL, E., GEYER, E. & 7 others (1977) Altered levels of cyclic nucleotides, cyclic AMP phosphodiesterase and adenylyl cyclase activities in normal, dysplastic and neoplastic human mammary tissue. *Febs Lett.*, **82**, 102.

MCNEILL, J. H., NASSAR, M. & BRODY, T. M. (1968) The effect of theophylline on amine-induced cardiac phosphorylase activation and cardiac contractility. *J. Pharmacol. Exp. Ther.*, **165**, 234.

MONAHAN, T. M., MARCHAND, N. W., FRITZ, R. R. & ABELL, C. W. (1975) Cyclic adenosine 3′,5′-monophosphate levels and activities of related enzymes in normal and leukemic lymphocytes. *Cancer Res.*, **35**, 2540.

PARDEE, A. B. (1974) A restriction point for control of normal animal cell proliferation. *Proc. Natl Acad. Sci. U.S.A.*, **71**, 1286.

PARKER, C. W. & SMITH, J. W. (1973) Alterations in cyclic adenosine monophosphate metabolism in human bronchial asthma. *J. Clin. Invest.*, **52**, 48.

PARKER, C. W., SULLIVAN, T. J. & WEDNER, H. J. (1974) Cyclic AMP and the immune response. *Adv. Cyclic Nucleotide Res.*, **4**, 1.

PASTAN, I. H., JOHNSON, G. S. & ANDERSON, W. B. (1975) Role of cyclic nucleotides in growth control. *Ann. Rev. Biochem.*, **44**, 491.

POLGAR, P., VERA, J. C., KELLEY, P. R. & RUTENBURG, A. M. (1973) Adenylate cyclase activity in normal and leukemic human leukocytes as determined by a radioimmunoassay for cyclic AMP. *Biochim. Biophys. Acta*, **297**, 378.

POLGAR, P., VERA, J. C. & RUTENBURG, A. M. (1977) An altered response to cyclic AMP stimulating hormones in intact human leukemic lymphocytes. *Proc. Soc. Exp. Biol. Med.*, **154**, 493.

PRASAD, K. N., KUMAR, S., BECKER, G. & SAHU, S. K. (1975) The role of cyclic nucleotides in differentiation of neuroblastoma cells in culture. In *Cyclic Nucleotides in Disease*. Ed. B. Weiss. Baltimore: University Park Press. p. 45.

PREUD'HOMME, J. L. & LABAUME, S. (1975) Immunofluorescent staining of human lymphocytes for the detection of surface immunoglobulins. *Ann. N.Y. Acad. Sci.*, **254**, 254.

RUDLAND, P. S., SEELEY, M. & SEIFERT, W. (1974) Cyclic GMP and cyclic AMP levels in normal and transformed fibroblasts. *Nature*, **251**, 417.

RYAN, W. L. & HEIDRICK, M. L. (1968) Inhibition of cell growth *in vitro* by adenosine 3′,5′-monophosphate. *Science*, **162**, 1484.

SALOMON, Y., LONDOS, C. & RODBELL, M. (1974) A highly sensitive adenylate cyclase assay. *Anal. Biochem.*, **58**, 541.

SANDLER, J. A., CLYMAN, R. I., MANGANIELLO, V. C. & VAUGHAN, M. (1975) The effect of serotonin (5-hydroxytryptamine) and derivatives on guanosine 3′,5′-monophosphate in human monocytes. *J. Clin. Invest.*, **55**, 431.

SCHER, N. S., QUAGLIATA, F., MALATHI, V. G., FAIG, D., MELTON, R. A. & SILBER, R. (1976) Cyclic adenosine 3′,5′-monophosphate phosphodiesterase activity in normal and chronic lymphocyte leukemia lymphocytes. *Cancer Res.*, **36**, 3958.

SCHULTZ, G., HARDMAN, J. G., SCHULTZ, K., BAIRD, C. E. & SUTHERLAND, E. W. (1973) The importance of calcium ions for the regulation of guanosine 3′:5′-cyclic monophosphate levels. *Proc. Natl Acad. Sci. U.S.A.*, **70**, 3889.

SCHWARZMEIER, J. D., LUJF, A., NEUMANN, E. & BÖHNEL, J. (1974) Zyklisches 3′,5′ Adenosinmonophosphat (cAMP) in normalen und PHA-stimulierten Lymphozyten sowie in leukämischen Zellen. *Wien. Klin. Wochschr.*, **86**, 8.

SEIFERT, W. E. & RUDLAND, P. S. (1974) Possible involvement of cyclic GMP in growth control of cultured mouse cells. *Nature*, **248**, 138.

SHEPPARD, J. R., GORMUS, R. & MOLDOW, C. F. (1977) Catecholamine hormone receptors are reduced on chronic lymphocytic leukaemic lymphocytes. *Nature*, **269**, 693.

SNEDECOR, G. W. (1962) *Statistical Methods.* Ames: Iowa State University Press. p. 318.

TAKEMOTO, D. J., LEE, W.-N. P., KAPLAN, S. A. & APPLEMAN, M. M. (1978) Cyclic AMP phosphodiesterase in human lymphocytes and lymphoblasts. *J. Cyclic Nucleotide Res.*, **4**, 123.

THOMPSON, W. J. & APPLEMAN, M. M. (1971) Multiple cyclic nucleotide phosphodiesterase activities from rat brain. *Biochemistry*, **10**, 311.

THOMPSON, W. J., ROSS, C. P., PLEDGER, W. J., STRADA, S. J., BANNERS, R. L. & HERSH, E. M. (1976) Cyclic adenosine 3′:5′-monophosphate phosphodiesterase. *J. Biol. Chem.*, **251**, 4922.

WATSON, J. (1975) The influence of intracellular levels of cyclic nucleotides on cell proliferation and the induction of antibody synthesis. *J. Exp. Med.*, **141**, 97.

WHITFIELD, J. F., MACMANUS, J. P., FRANKS, D. J., GILLAN, D. J. & YOUDALE, T. (1971) The possible mediation by cyclic AMP of the stimulation of thymocyte proliferation by cyclic GMP. *Proc. Soc. Exp. Biol. Med.*, **137**, 453.

YANG, T. J. & VAS, S. I. (1971) Growth inhibitory effects of adenosine 3′,5′-monophosphate on mouse leukaemia L-5178-Y-R-cells in culture. *Experientia*, **27**, 442.